# A simple, generalizable method for measuring individual research productivity and its use in the long-term analysis of departmental performance, including between-country comparisons

**DOI:** 10.1186/1478-4505-11-2

**Published:** 2013-01-14

**Authors:** Richard Wootton

**Affiliations:** 1 Norwegian Centre for Integrated Care and Telemedicine, University Hospital of North Norway, PO Box 6060, Tromsø N-9038, Norway; 2 Faculty of Health Sciences, University of Tromsø, Tromsø N-9037, Norway

**Keywords:** Research productivity, Research output, Capacity building

## Abstract

**Background:**

A simple, generalizable method for measuring research output would be useful in attempts to build research capacity, and in other contexts.

**Methods:**

A simple indicator of individual research output was developed, based on grant income, publications and numbers of PhD students supervised. The feasibility and utility of the indicator was examined by using it to calculate research output from two similarly-sized research groups in different countries. The same indicator can be used to assess the balance in the research “portfolio” of an individual researcher.

**Results:**

Research output scores of 41 staff in Research Department A had a wide range, from zero to 8; the distribution of these scores was highly skewed. Only about 20% of the researchers had well-balanced research outputs, with approximately equal contributions from grants, papers and supervision. Over a five-year period, Department A's total research output rose, while the number of research staff decreased slightly, in other words research productivity (output per head) rose. Total research output from Research Department B, of approximately the same size as A, was similar, but slightly higher than Department A.

**Conclusions:**

The proposed indicator is feasible. The output score is dimensionless and can be used for comparisons within and between countries. Modeling can be used to explore the effect on research output of changing the size and composition of a research department. A sensitivity analysis shows that small increases in individual productivity result in relatively greater increases in overall departmental research output. The indicator appears to be potentially useful for capacity building, once the initial step of research priority setting has been completed.

## Background

Appropriate research capacity is an essential component of any public health system. Consequently, building research capacity is a common aim at an institutional level, as well as at national and international levels. In order to inform resource allocation decisions, an agreed method of measuring research capacity would be desirable. This in turn implies some method for measuring “research” itself, either research impact or research output. Unfortunately, although measuring the impact of research is possible, it is not straightforward [1,2]; measuring research output is in many ways an easier problem and a wide range of methods has been proposed for this purpose. Some have depended solely on publication output [3], while others have taken account of indices such as research grant income and administrative service commitments [4]. Nevertheless, none have achieved widespread acceptance. The intention of all such measures is to provide management information; that is, information which can be used to assess the performance of individual researchers, or groups of researchers. The assessments may be cross-sectional, for example to compare productivity between organizational units, or they may be longitudinal, such as to examine the variation in the performance of an individual researcher over time.

How then do we measure research productivity? There is a wide range of indicators and metrics which might be relevant. Indicators ‘indicate’ impact, but they do not attempt to quantify it, while metrics are ‘numerical indicators’ that allow the impact to be quantified. In the present context, measuring the impact of research is too large a task; see Banzi *et al.*[5] for an overview of reviews. Thus the present paper concerns the preceding step, the measurement of research output. This raises the question, what do we mean by research output? Research output is the product of performing research activities. Thus it may include: i) publishing: writing papers (peer-reviewed and non-peer-reviewed), book chapters, books, popular articles; ii) gaining grants: competitive (peer-reviewed) and non-competitive; iii) supervising research students (PhDs and others); iv) serving as a peer-reviewer (of grants and papers), examining PhDs, acting as a member of an editorial board; v) giving lectures and other presentations, especially as an invited (keynote) speaker; vi) contributing to national and international committees, for example to produce practice guidelines; and vii) filing patents. This list is not exhaustive.

### Choice of indicator variables

What variables should be used to measure research output? Clearly there is a very large number of possible indicator variables. However, equally obviously, there is a tradeoff: as the complexity of the indicator increases, so it becomes more difficult to collect the requisite data. Furthermore, there is less likelihood of generality, since there will be less agreement about what are the proper constituent variables and how they are defined. For example, what constitutes a prestigious lecture at an important meeting? A complex indicator may be better tailored to a particular local environment, but almost by definition will become less generally applicable.

The literature contains many reports of research productivity, in most of which research output has been based on publications, usually peer-reviewed papers. Some studies have also taken into account grant income. A number of these studies are summarized in Table 1. Of the twelve studies listed, seven assessed research performance at the level of individual researchers, two studies assessed it at departmental level, and four assessed it at the institutional level. The period over which data were collected ranged from one to ten years.

**Table 1:** Studies in which research productivity was assessed

**Study**	**Level of assessment**	**Country**	**indicator variables**^1^	**Sampling epoch**^2^
Brocato 2005 [10]	Individual	US	peer-reviewed journal articles	2 years
	474 faculty members in family medicine departments		national conference presentations	
			national grants	
Cox 1977 [11]	Institutional	US	papers in the APA journals	5 years
	76 graduate programs in psychology			
Dakik 2006 [12]	Individual	Lebanon	journal papers (Medline articles only)	5 years
	203 medical faculty staff at a university in Beirut			
Ellwein 1989 [3]	Individual and Departmental	US	journal papers	3 years
	Faculty members in 12 departments of a university medical faculty			
Howard 1987 [6]	Institutional	US	papers in APA journals	10 years
	75 institutions granting doctoral degrees in psychology			
Iyengar 2009 [13]	Individual	US	journal papers	2 years
	158 individual faculty members of a school of medicine		grant income	
			research space used	
Kranzler 2011 [14]	Institutional	US	papers from PsycInfo database	5 years
	59 institutions offering doctoral programs in school psychology			
Mezrich 2007 [4]	Individual	US	peer-reviewed papers	1 year
	Members of a large university radiology department		other publications	
			grants	
Salaran 2010 [15]	Individual	Australia	books (single or multi-author)	3 years
	271 academic staff in 5 universities in Melbourne		papers in refereed journals	
			edited books	
			chapters in refereed books	
Tien 1996 [16]	Individual	US	publications	2 years
	2586 faculty members in US universities			
Toutkoushian 2003 [17]	Institutional	US	publications (ISI)	1 year
	1309 colleges and universities			
Wagner 1994 [18]	Departmental	US	publications in 6 family medicine journals	5 years
	Family medicine departments			

^1^In some studies publication credit was adjusted to take account of the journal Impact Factor and/or the author position in articles with multiple authors.

^2^Period over which data were collected. Some studies collecting data over 5 years reported it for shorter periods.

Based on my own experience of being assessed by promotion committees and Deans of medicine, a research output measurement based only on publications and grant income would be considered too narrow. A credible researcher would be expected to undertake PhD supervision. Other factors, such as writing book chapters or national committee work, would certainly be assumed to occur, but only their absence would be remarked upon. My hypothesis is that a useful indicator of research output can be constructed, based on three variables.

## Methods

A simple and generalizable indicator of research output is defined. This is based on data relating to individual researchers. The feasibility and utility of the indicator is examined in a number of different settings. This is followed by a discussion about its interpretation. The three indicator variables are: i) research grant income; ii) peer-reviewed publications; and iii) PhD students supervised. Activity in each domain is converted to points, which are used to calculate a score for research output. The sampling epoch is one calendar year by default.

### Grants

Credit is given for research grants: i) which have been awarded competitively; ii) with funding for all or part of the year in question. These are weighted by grant-holder positions (see the section on *Name Position* below). Grant income is used to calculate grant “points”. The grant income is that proportion of the grant which is payable during the year in question. Within a given country, the currency unit of that country can be scaled appropriately in order to calculate these points, for example in Norway, where research grants are commonly of the order of millions of Kroner (NOK), a convenient scaling factor is 1 million NOK = 1 grant point. To facilitate comparisons between countries, where different currencies may be in use, different currency scaling factors are required. This matter is considered further below.

### Papers

Credit is given for papers: i) which are listed in Medline (strictly, listed in PubMed), in other words, peer-reviewed papers; ii) which have been published during the year in question (note that the date of publication is available in Medline). These are weighted by the journal Impact Factor (using Impact Factors from a standard source, such as JCR Web) and also weighted by author position (see the section on *Name Position* below). Peer-reviewed papers are used to calculate publication “points”.

### PhD students

Credit is given for supervising PhD students: i) where the subject matter of the thesis is relevant to the research interests of the organization; ii) where the supervision occurs during all or part of the year in question. The supervision must be registered with the relevant university. Note, however, that no credit is given after the due date for completion, usually three years from the date of registration. PhD supervision is used to calculate PhD “points”.

### Research output score

The research output score is the sum of grant points (g), publication points (p) and PhD supervision points (s):

R=g+p+s

For simplicity, these variables are given equal weighting, although obviously other weighting schemes could be used, as required.

### Dimensionality

The research output score as described allows comparison within an organizational unit, for example from year to year. It also allows comparison between units in the same country. However, there is a problem with comparison between units from different countries, because the grant income is measured in units of the national currency. Publication points and student points are dimensionless, being composed of factors such as ratios (such as journal impact factor and author credit) and pure numbers. Grant points, as described above, have the units of the national currency in which grants are awarded. Therefore direct comparisons between countries are not possible without non-dimensionalization of the grant points. This can be achieved by adjusting the reported grant income for purchasing power in the country concerned. As suggested above, a currency scaling factor can be used, which is loosely based on the perceived value of grants. However, this is a subjective decision. Furthermore, currency scaling factors are likely to change from year to year as a result of inflation.

A more rigorous method would be to express grant income in multiples of some common amount, the amount being meaningful in all countries conducting medical research. One possibility would be to use the national research council’s standard award for a PhD studentship. For example, at the time of writing, the Norwegian Research Council offers three-year studentships at 877,000 NOK/year. Thus grant income in that country would be divided by 877,000. This normalizes the grant points between countries and simultaneously deals with the problem of inflation.

### Name position

The order of authors’ names on a paper has a deep and mystical significance. Everyone agrees that the order matters, although there is no consensus about the details. Howard *et al.* proposed a monotonic scheme of assigning author credit in multi-author papers [6]. The weights (credit) for authors on papers with up to five authors are shown in Table 2A.

**Table 2:** Schemes for assigning credit to authors of multi-author papers

**No. of authors**	**First author**	**Second**	**Third**	**Fourth**	**Fifth**
(A) Monotonic scheme proposed by Howard *et al.*[6].					
1	1.0000				
2	0.6000	0.4000			
3	0.4737	0.3158	0.2105		
4	0.4154	0.2769	0.1846	0.1231	
5	0.3839	0.2559	0.1706	0.1137	0.0758
(B) FLAE scheme proposed by Tscharntke *et al.*[8].					
1	1.0000				
2	0.66667	0.33333			
3	0.54545	0.18182	0.27273		
4	0.50000	0.12500	0.12500	0.25000	
5	0.47619	0.09524	0.09524	0.09524	0.23810
(C) FLAE scheme proposed by Ellwein *et al.*[3].					
1	1.0000				
2	0.5556	0.4444			
3	0.3846	0.3077	0.3077		
4	0.3086	0.2469	0.1975	0.2469	
5	0.2577	0.2062	0.1649	0.1649	0.2062
(D) FLAE scheme proposed in the present study. For more than two authors, the first author is assigned 50% of the credit, the last author 30%, and the also-rans have the remaining 20% divided between them.					
1	1.0000				
2	0.7000	0.3000			
3	0.5000	0.2000	0.3000		
4	0.5000	0.1000	0.1000	0.3000	
5	0.5000	0.0667	0.0667	0.0667	0.3000

While this scheme has the merit of simplicity, it does not reflect the common perception in medicine that the last author of a multi-author paper has performed a group leadership role and should receive almost as much credit as the first author [7]. This can be taken into account in a “first-last-author-emphasis” or FLAE scheme, such as that proposed by Ellwein *et al.*[3] or Tscharntke *et al.*[8], see Table 2B,C. A similar scheme, which is slightly less complicated to calculate, is shown in Table 2D. For simplicity, the same weighting scheme is used in the present work for both papers and grants.

### Feasibility, validity and utility

The feasibility of the research output score was examined by using it to calculate the research output from two research departments, in different countries. The two groups are of similar size. Each group conducts medical research in association with a university teaching hospital.

The validity of the score was examined by comparing it with an independent expert assessment of the research output of the members of one of the departments. The assessment was made by the recently-retired head of the department (and ex-Dean of the Faculty) who rated each researcher, based on their publications and research performance generally. A five-point scale was used (1 = very good; 2 = good; 3 = average; 4 = poor; 5 = very poor).

The utility of the research output score was examined in three different (national and multinational) settings: i) individual comparison within a research group for a particular year; ii) longitudinal comparison of a research group from year to year; iii) comparison between two research groups for a particular year.

## Results

### Feasibility

There were no conceptual difficulties in using the simple measure proposed. In practice, obtaining accurate data on publications, grants and PhD supervision was straightforward, although performing the calculations manually was very tedious. A web-based calculator was therefore written to automate the process.

### Validity

**Figure 1:**
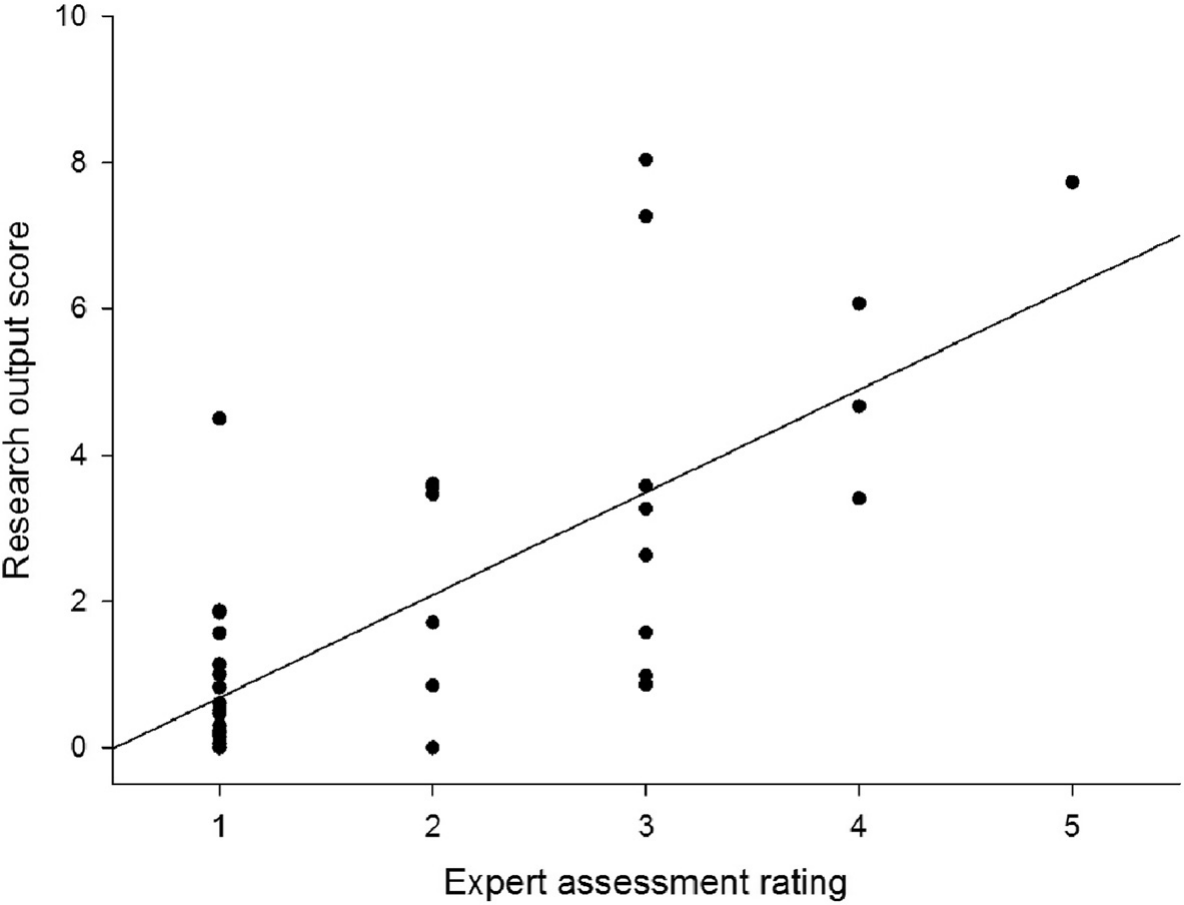
**Independent expert assessment and research output scores. **The expert assessment ratings are shown reversed for clarity (1 = worst; 5 = best).

There was a significant correlation (r = 0.71) between the independent expert assessment of the researchers’ performance and their research output scores, Figure 1. A formal method comparison was not attempted as the output scores are unbounded.

### Utility in different settings

The utility of the proposed measure was examined in different settings to confirm that the results appeared plausible and potentially useful for management purposes.

#### Within-group comparison

**Figure 2:**
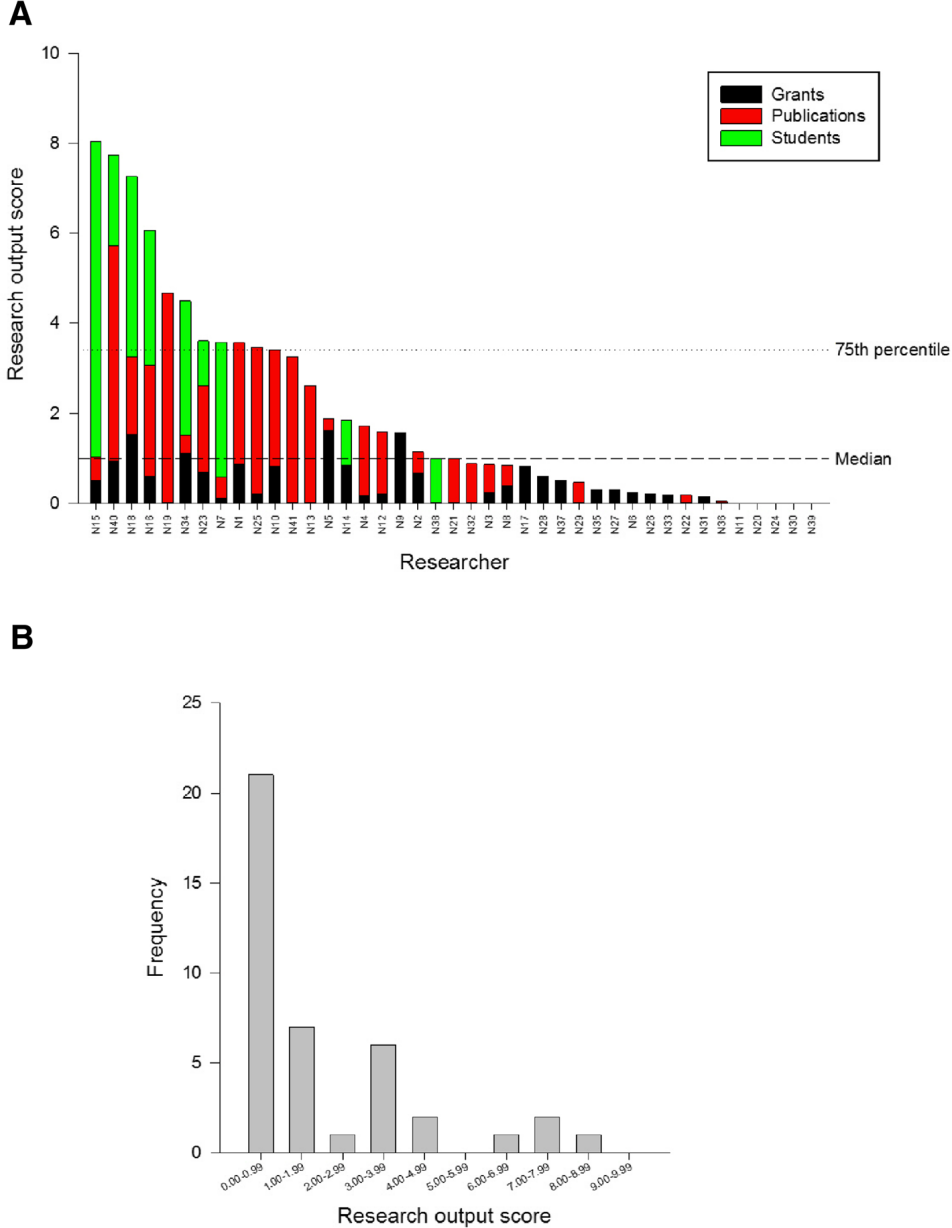
Research output scores (in 2011) for the individual members of Research Department (A); the frequency distribution of scores (B).

Research Department A has been in existence for about seven years. Research output scores for its individual members, calculated as described above, are shown in Figure 2A. The output scores range from 0 to 8. The frequency distribution is positively skewed (Figure 2B). The output scores reflect the perception that Department A contains a few research-active individuals and a larger number of research-inactive staff.

#### Longitudinal comparison

**Figure 3:**
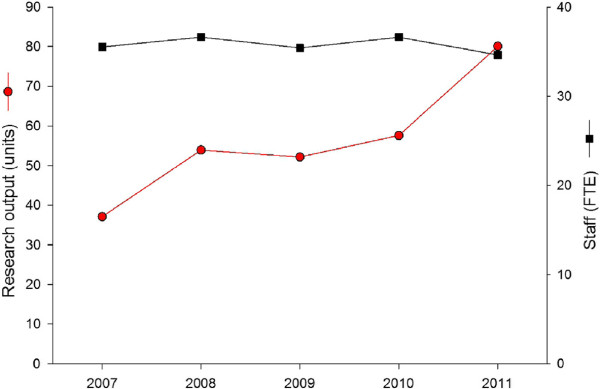
Research productivity and staff size in successive years (same currency scaling factor each year) for Research Department A.

Research Department A contains about 40 research staff. The total research output score for the department can be calculated as described above. In 2011, the total research output of this research department was 80.1 units. This can be normalized to provide an average output score per staff member, which was 2.3 units/full-time equivalent (FTE). Over a period of several years, the number of staff has fluctuated, as a result of arrivals and departures. The trend in the Department’s output score, see Figure 3, reflects the perception that although staff numbers have decreased slightly, research productivity (output per head) has risen.

#### Between group comparison

**Figure 4:**
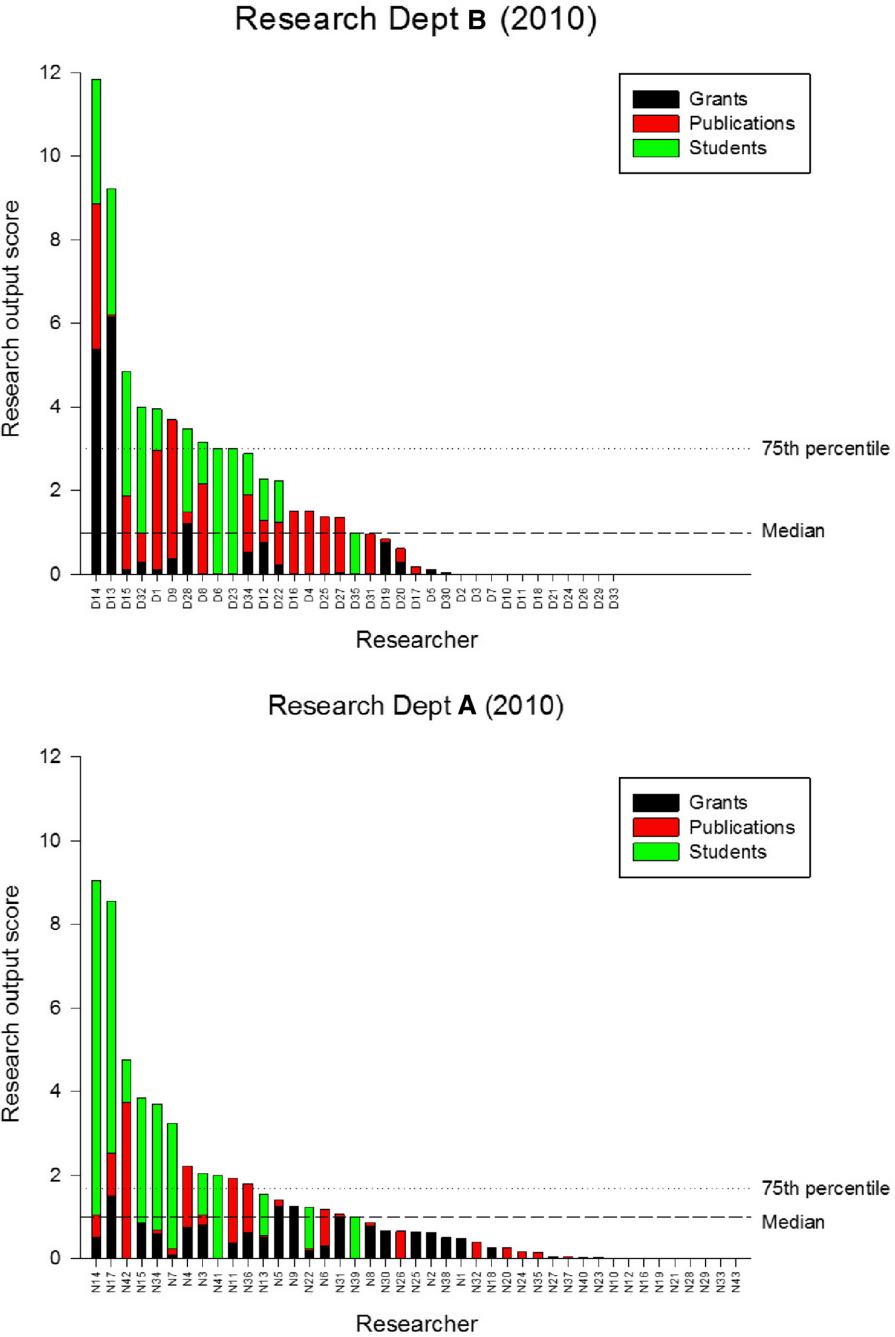
Between group comparison.

Research Department B is of approximately the same size as Department A, but it is located in a different country with a different currency. A currency scaling factor was chosen (£100,000 = 1 grant point) based on the total grant income of the two departments. Research Department B had a total research output score of 67.1 in 2010, which was somewhat higher than the research output of Research Department A, see Table 3. The pattern of individual research output scores was similar in the two departments, in other words, a small number of individuals with high scores and a proportion with low or zero scores, see Figure 4.

**Table 3:** Research output of two groups based in different countries for the same year (2010)

	**Staff (FTE)**	**Total grant income**	**Currency scaling factor (per grant point)**	**Research output**	**Mean research output (per FTE)**
Dept A	36.6	14.6 million NOK	1 million NOK	57.6	1.6
Dept B	34.0	£1.57 million	£100,000	67.1	2.0

### Portfolio balance

As can be seen in Figure 2A, real-life data from a research department with 41 staff show that some individuals have a research output based approximately equally on grants, publications and supervision. On the other hand, some individuals have a research output based only on publications, or only on grants, or only on supervision. It can be assumed that a well-rounded, mature researcher would not exhibit the latter patterns. Indeed, it seems reasonable to expect that the research output of a well-developed and successful researcher would comprise in approximately equal parts, publications, grants and PhD supervision. How can this “balance” in the portfolio be measured? One simple method is to calculate the variance in the three components, i.e. the greater the difference between the values of the three points, the higher the variance. To facilitate comparison between individuals with different research outputs, the variance can be normalized, the simplest method being to compute the coefficient of variation (CV) or relative standard deviation.

**Figure 5:**
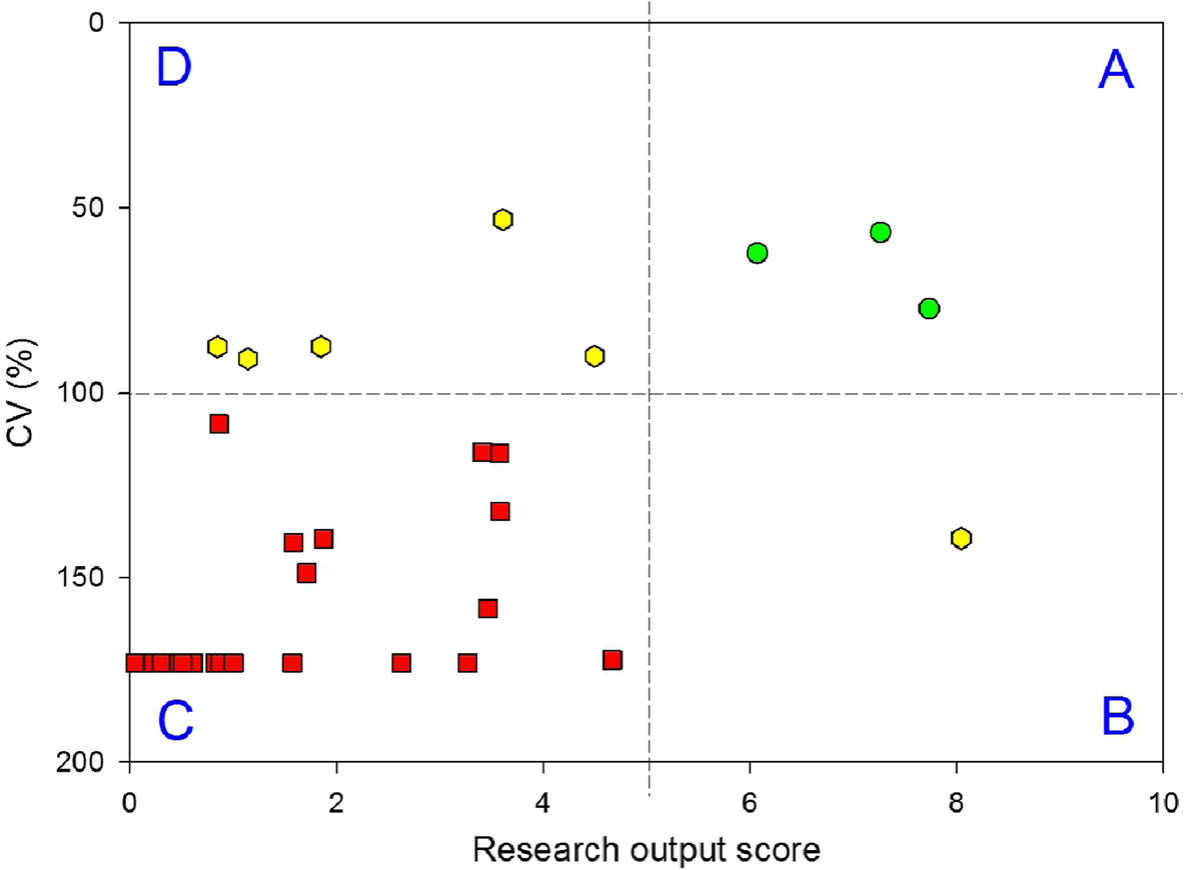
**Research output and portfolio balance (CV of the constituent scores) for Research Department A in 2011. **The most successful researchers lie in quadrant **A**, with a high research output and a low CV, therefore a well balanced portfolio (circles). The least successful researchers lie in quadrant **C** (squares).

Using the dataset of Figure 2A indicates that some individuals have a well balanced portfolio of research output, with CVs below 100%, while others have much more disparate scores resulting in CVs of nearly 200%. This information represents a useful managerial tool, which can potentially be used to influence research performance. Figure 5 shows the output scores of the researchers plotted against the CV in their scores (note, CV is plotted on a reverse scale). Thus the best researchers in this group fall into the upper right quadrant (A), with a high research output and a low CV; those in the other quadrants are less successful in one or more ways. There are four researchers with high output. However, one of them is mainly supervising PhD students, which is responsible for nearly 90% of that person’s output score. Perhaps this individual needs to be encouraged to publish more and to seek additional grants.

## Discussion

Those who fund research and those who influence funding decisions, would ideally like to measure the impact of that research. In practice, this is not straightforward and there have been few attempts to make comprehensive assessments of research impact in health-related R&D. Research output is undoubtedly a weak proxy for research impact, but is arguably better than nothing. The present paper proposes a simple method of measuring research output.

In the measurement of research output, the perspective of the assessor may be a factor to be considered. For example, different views may be taken by the head of a research unit, the head of an organizational unit (the Dean or the Vice Chancellor, say), by the funding body concerned (national research council, for example) or by the researchers’ peers. These people may also take different views about the unit of assessment, which could be faculty members, a center, a department, a research group or an individual researcher. Nonetheless, the present paper shows that a single assessment system may suffice.

### Assumptions

Calculation of the research output score rests on a number of assumptions. These include: i) Grants and papers: a method of assigning credit to authors of multi-author work is required, and there is no generally agreed system for doing this. The present paper proposes a simple system; ii) Grants: if two otherwise identical grants are made for the same piece of work at different institutions, the amount of the award will not necessarily be the same. For example, institutions in capital cities often have higher overheads. Thus the research credit for the grant will not be the same; iii) Papers: the journal Impact Factor has many well-known drawbacks. On the other hand, it is widely used in medicine. In the proposed indicator, non-Medline papers are ignored, largely for convenience. Publications in non-Medline journals are therefore considered in the same way as books and reports, i.e. worthy output, but not readily quantifiable for present purposes; iv) PhD supervision: supervision of research students is only one form of postgraduate teaching. On the other hand, it is an important part of research output; v) Weighting: in the proposed indicator, equal weight is given to grant income, publications and students supervised. That is, in assessing research output, the implication is that a grant of 1 million NOK is worth as much to the organization as a single-author paper in a journal with IF = 1. In the absence of a case for something more complicated, equal weighting seems appropriate; vi) Research output components: a well-balanced portfolio is desirable. I am not aware of any published work on this topic; vii) Currency scaling factor (or other normalizing factor): an appropriate factor must be used if scores are to be compared between countries.

### Leavers and joiners

The research output score is calculated for a particular period: one year by default. During this sampling period, staff turnover may occur. To adjust for staff who leave or join during the year in question, a “year_fraction” can be calculated, for example a researcher who leaves on the 30th of June would have been in post for six months of the year in question. The year_fraction would therefore be 0.5, and the person’s research output can be adjusted by this factor. However, in practice it is difficult to arrive at a sensible adjustment scheme: a researcher who leaves after six months could reasonably be supposed to have an adjusted output twice as high; but a researcher who leaves after six days … should the adjusted output be 365/6, i.e. approximately 60-times as high? Ellwein *et al.*[3] pointed out that partial calendar years of employment could reasonably be treated as full years, since all publications from any one calendar year were included. For the present purposes, staff employed for part of the year in question are treated as though they had worked for the whole year.

### Part-time staff

The calculation of research output as described above relates to full-time staff. Researchers may, however, be employed on a part-time basis, such as one day per week. In principle, their research output could be adjusted to provide a more equitable basis for comparison with full-time staff. Suppose a researcher is employed on a one day per week contract. That researcher’s output could be adjusted to reflect the fact that the rest of the staff in the department are employed to work five days per week. So, for grants awarded and papers published, perhaps the part-time researcher should receive greater credit: their output could be multiplied by some factor, perhaps by five. The position with PhD supervision is debatable. On balance it seems that most one-day-a-week researchers would be likely to be supervising students in their four-day-a-week “proper” jobs. So perhaps the credit for PhD supervision should be reduced by some factor, perhaps by five.

The factor used to take account of the output from part-time staff could be in linear relation to their employment, for example the output from a 20% person is multiplied by 5, the output from a 10% person is multiplied by 10, and so on. In practice, this seems to be too heavily weighted in favor of the part-timers. A damped weighting scheme could therefore be employed, where the weighting is inversely proportional to the square root of the FTE. However, in practice this adjustment does not seem worthwhile.

### Other adjustments

There is a common perception that publishing is more difficult in certain research fields, depending on the numbers of journals and the perceived academic prestige of that field. In principle, adjustments could be made to take account of the different impact factors of journals in different research fields. However, in practice the median journal impact factors, as listed in JCRWeb, are not very different between research fields. For example, the field of Medicine (General and Internal) has 153 journals with a median impact factor of 1.104. In contrast, telemedicine is a much smaller field, with only two specialist journals. However, their median impact factor is very similar, at 1.286.

Taking all this into account, it seems unnecessary to make adjustments for staff turnover, for part-time workers and for publishing in different research fields.

### Interpretation

Calculation of the simple score described above is feasible in practice. A comparison with the assessments made by an expert suggests that the scores are valid and reflect reality, although the absence of a gold standard precludes absolute proof being obtained. Furthermore, the scores appear to provide useful information in a general sense, such as at departmental level and above. However, in employing the score as a capacity-building tool, what are we aiming for? What do we expect from the ideal research group? Clearly this will depend on the structure and composition of that research group.

A research group of any reasonable size is likely to contain researchers at different stages of their careers. Plainly neophyte researchers, such as early-stage PhD students, could not be expected to have significant research output, while late-stage researchers, such as heads of department, may no longer be as active in publishing and grant-winning as they were in former days; between these extremes, one would expect higher personal research output. How is departmental research output influenced by the structure and composition of the staff? How is output influenced by changes in work practices, such as increased publication rates or higher grant income? These questions can be answered by modeling.

### Modeling

To model the output from a hypothetical research group, its size and composition must be chosen first. The following example concerns a research group of similar size to Research Department A. It is composed of a departmental head and three subgroups, each with 13 staff and one group leader; 40 staff in total. Each subgroup comprises a senior researcher as group leader, supported by two mid-rank researchers and four post-doctoral fellows. There are six PhD students in each subgroup, three in the first half of their studentships and three in the second half. See Table 4A.

**Table 4:** Baseline model

**(A): Structure**								
							**No. in the Dept**	
Head of Dept	1		1				1	
	**Group A**		**Group B**		**Group C**			
Group leader	1		1		1		3	
Mid-rank researcher	2		2		2		6	
Post-doctoral fellow	4		4		4		12	
PhD student (junior)*	3		3		3		9	
PhD student (senior)**	3		3		3		9	
*Total*	*13*		*13*		*13*		*40*	
*First half of PhD studentship; **Second half of PhD studentship								

**(B): Grants. The total grant income for the Department is 16.8 million NOK**								
	**Grant income per person (NOK /yr)**				Grant points per person*			
Head of Dept	250,000				0.250			
Group leader	500,000				0.500			
Mid-rank researcher	1,000,000				1.000			
Post-doctoral fellow	750,000				0.750			
PhD student (junior)	0				0.000			
PhD student (senior)	0				0.000			
*Grant points = grant income multiplied by the currency scaling factor (1 million NOK = 1 point)								

**(C): Papers. The total number of papers published by the Department is 91**								
	No. of papers per person, a*		**Author-position/ Type of paper**	Author score per paper, b*		Journal impact factor, c*	Publication points per person*	
Head of Dept	1		AU 6 of 6	0.300		1.5	0.450	
Group leader	2		AU 3/4/5 of 6	0.050		1.5	0.150	
Mid-rank researcher	3		AU 2 of 3	0.200		1.5	0.900	
Post-doctoral fellow	4		AU 3 of 3	0.300		1.5	1.800	
PhD student (junior)	0		-	-		-	0.000	
PhD student (senior)	2		First AU of 2	0.700		1.5	2.100	
*Publication points = a x b x c								

**(D): PhD students. The total number of PhD students supervised by members of the Department is 18**								
	**PhD students per person**				PhD points per person*			
Head of Dept	0				0			
Group leader	1				1			
Mid-rank researcher	1.5				1.5			
Post-doctoral fellow	0.5				0.5			
PhD student (junior)	0				0			
PhD student (senior)	0				0			
*PhD points = no of students supervised								

**(E): Points. Scores, per individual and total for the Department**								
	**Total score per individual**	Portfolio balance (%)*	**No. of individuals**	**Total grant points**	**Total publication points**	**Total PhD points**	**Total score**	**Score% of Dept total**
Head of Dept	0.700	97	1	0.250	0.450	0.000	0.700	1
Group leader	1.650	78	3	1.500	0.450	3.000	4.950	6
Mid-rank researcher	3.400	28	6	6.000	5.400	9.000	20.400	25
Post-doctoral fellow	3.050	68	12	9.000	21.600	6.000	36.600	45
PhD student (junior)	0.000	-	9	0.000	0.000	0.000	0.000	0.0
PhD student (senior)	2.100	173	9	0.000	18.900	0.000	18.900	23
*Total*			*40*	*16.750*	*46.800*	*18.000*	*81.550*	*100.0*
*Portfolio balance is the coefficient of variation of the three component scores. Lower values indicate more uniformity								

To calculate the research output expected from this department, the grant income, publications and PhD supervision of the members must then be assumed. What sort of publication output is it reasonable to expect? Publication output per staff member has been measured in a number of studies, most of which were conducted in the US. A range of publication outputs was reported, from 0.9 to 4.9 papers/person/year (Table 5). The mean publication rate was about 2 papers/person/year. I am not aware of similar quantitative data relating to grant income or PhD supervision which is generally available.

**Table 5:** Estimates of individual publication rate

**Study**	**Sample**	**Data source**	**No.**	**Mean (papers/person/year)**	**SD**
Brocato 2005 [10]	Faculty members from family medicine departments in the US	Peer-reviewed journal articles	474	0.95	1.43
Dakik 2006 [12]	Medical faculty at a university in Beirut	Medline papers	203	1.24	1.38
Ellwein 1989 [3]	Medical faculty at a university in Nebraska	Published papers	372	1.95	N/A
Kranzler 2011 [14]	Faculty members of US doctoral programs in school psychology	Peer-reviewed papers in PsycINFO database	59	4.93	3.38
Tien 1996 [16]	Health faculty members in US universities	Self-reported publications			
	assistant professors		27	0.97	0.93
	associate professors		37	1.88	2.13
	full professors		23	2.18	1.62
Toutkoushian 2003 [17]	All faculties at US universities and colleges (data presented for top 50 only)	ISI databases	50	2.86	1.30
Wagner 1994 [18]	Faculty members from family medicine departments in the US	Six important family medicine journals	21	0.91	0.70

The weighted mean is 1.6 publications/person/year.

Taking this into account, the grant incomes expected for each grade of staff are shown in Table 4B. The total grant income for the department is 16.75 million NOK. The publications per member of staff are shown in Table 4C, assuming relatively modest author contributions and journal impact factors. Finally, the PhD supervision of each grade of staff are shown in Table 4D. Based on this, the research output from this hypothetical department amounts to 81.6 units, Table 4E. That is, it is of the same order as the actual research outputs from Departments A and B.

**Figure 6:**
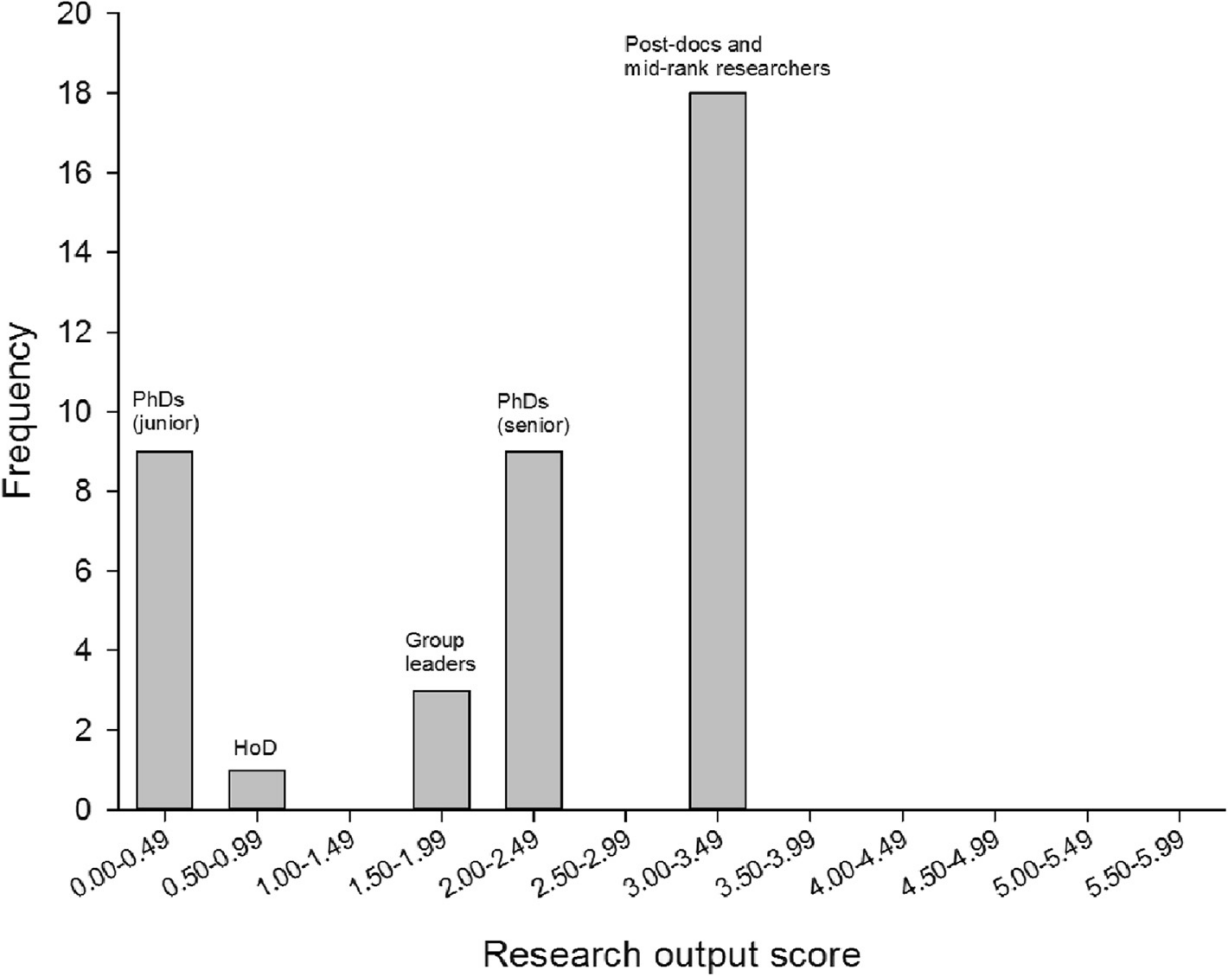
Distribution of research output scores in the baseline model (n = 40).

The distribution of the individual research output scores from the baseline model is shown in Figure 6. This shows a U-shaped distribution, rather different from the skewed distribution of Research Department A (Figure 2B). In other words, the baseline model has relatively fewer individuals with zero or low output scores, and relatively more with high output scores. Inspection of Figure 6 shows that the only staff with zero scores are the early-period PhD students, while the post-doctoral fellows and the mid-rank researchers represent the “engine room” of research output, being responsible for two-thirds of the department’s total output.

Modeling can be used to explore the effect on research output of changes in staffing and performance.

#### Sensitivity analysis

An elementary sensitivity analysis can be used to examine the effect of changes in staff productivity on the research output from the department. For example, all members of staff could in principle increase their productivity by 10%. That is, their individual grant income could be increased by 10%, the numbers of papers published could be increased by 10%, and the number of PhD students supervised could be increased by 10%. In these circumstances the output from the department would increase: grant income from 16.8 million NOK to 18.4 million NOK, papers from 91 to 100, and PhD students supervised from 18 to 20. The net effect is that total research output would increase from 81.6 to 100.5 units.

**Figure 7:**
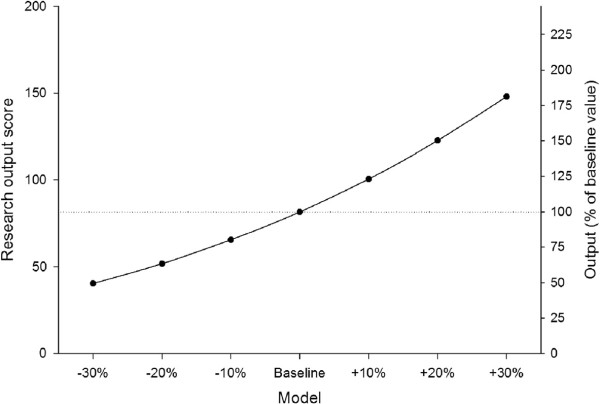
Sensitivity analysis.

Figure 7 shows the effect on departmental research output of changes in staff productivity. There is a non-linear relationship (in fact, cubic), so that small increases in individual staff productivity produce larger increases in total research output.

## Conclusions

In developing a method for measuring research output it must be recognized that there is no right answer: there are different metrics for different circumstances. The guiding principles should be to keep things simple and to state the assumptions clearly. The present work demonstrates the feasibility, utility and apparent validity of a simple and generalizable method of measuring research output. The output score is dimensionless and can be used for comparisons within and between countries. It can therefore be employed in capacity building, following the initial step of research priority setting [9].

The methods described can be used to inform resource allocation, helping to ensure that money is spent wisely, fairly and efficiently. This is true of capacity building in resource-limited settings, and more generally in the industrialized world as well.

## Competing interests

The author declares that he has no competing interests.

## References

[B1] Lavis J Ross S McLeod C Gildiner A Measuring the impact of health research J Health Serv Res Policy 2003 8 165 170 10.1258/135581903322029520 12869343

[B2] Croxson B Hanney S Buxton M Routine monitoring of performance: what makes health research and development different? J Health Serv Res Policy 2001 6 226 232 10.1258/1355819011927530 11685787

[B3] Ellwein LB Khachab M Waldman RH Assessing research productivity: evaluating journal publication across academic departments Acad Med 1989 64 319 325 10.1097/00001888-198906000-00008 2719791

[B4] Mezrich R Nagy PG The academic RVU: a system for measuring academic productivity J Am Coll Radiol 2007 4 471 478 10.1016/j.jacr.2007.02.009 17601589

[B5] Banzi R Moja L Pistotti V Facchini A Liberati A Conceptual frameworks and empirical approaches used to assess the impact of health research: an overview of reviews Health Res Policy Syst 2011 9 26 10.1186/1478-4505-9-26 21702930 PMC3141787

[B6] Howard GS Cole DA Maxwell SE Research productivity in psychology based on publication in the journals of the American Psychological Association Am Psychol 1987 42 975 986

[B7] Wren JD Kozak KZ Johnson KR Deakyne SJ Schilling LM Dellavalle RP The write position, A survey of perceived contributions to papers based on byline position and number of authors EMBO Rep 2007 8 988 991 10.1038/sj.embor.7401095 17972896 PMC2247376

[B8] Tscharntke T Hochberg ME Rand TA Resh VH Krauss J Author sequence and credit for contributions in multiauthored publications PLoS Biol 2007 5 e18 10.1371/journal.pbio.0050018 17227141 PMC1769438

[B9] Tomlinson M Chopra M Hoosain N Rudan I A review of selected research priority setting processes at national level in low and middle income countries: towards fair and legitimate priority setting Health Res Policy Syst 2011 9 19 10.1186/1478-4505-9-19 21575144 PMC3115910

[B10] Brocato JJ Mavis B The research productivity of faculty in family medicine departments at U.S. medical schools: a national study Acad Med 2005 80 244 252 10.1097/00001888-200503000-00008 15734806

[B11] Cox WM Catt V Productivity ratings of graduate programs in psychology based on publication in the journals of the American Psychological Association Am Psychol 1977 32 793 813

[B12] Dakik HA Kaidbey H Sabra R Research productivity of the medical faculty at the American University of Beirut Postgrad Med J 2006 82 462 464 10.1136/pgmj.2005.042713 16822923 PMC2563770

[B13] Iyengar R Wang Y Chow J Charney DS An integrated approach to evaluate faculty members’ research performance Acad Med 2009 84 1610 1616 10.1097/ACM.0b013e3181bb2364 19858825

[B14] Kranzler JH Grapin SL Daley ML Research productivity and scholarly impact of APA-accredited school psychology programs: 2005–2009 J Sch Psychol 2011 49 721 738 10.1016/j.jsp.2011.10.004 22272794

[B15] Salaran M Research productivity and social capital in Australian higher education High Educ Quarterly 2010 64 133 148 10.1111/j.1468-2273.2009.00448.x

[B16] Tien FT Blackburn RT Faculty rank system, research motivation, and faculty research productivity J High Educ 1996 67 2 22 10.2307/2943901

[B17] Toutkoushian RK Porter SR Danielson C Hollis PR Using publications counts to measure an institution’s research productivity Res High Educ 2003 44 121 148 10.1023/A:1022070227966

[B18] Wagner PJ Hornsby JL Talbert FS Hobbs J Brown GE Kenrick J Publication productivity in academic family medicine departments Fam Med 1994 26 366 369 8050658

